# Integrating natural compounds and nanoparticle‐based drug delivery systems: A novel strategy for enhanced efficacy and selectivity in cancer therapy

**DOI:** 10.1002/cam4.7010

**Published:** 2024-03-16

**Authors:** Asma Manzari‐Tavakoli, Amirhesam Babajani, Maryam Manzari Tavakoli, Fahimeh Safaeinejad, Ameneh Jafari

**Affiliations:** ^1^ Department of biology faculty of science Ferdowsi University of Mashhad Mashhad Iran; ^2^ Oncopathology Research Center, Department of Molecular Medicine, School of Medicine Iran University of Medical Sciences Tehran Iran; ^3^ Department of Phytochemistry Medicinal Plants and Drugs Research Institute, Shahid Beheshti University Tehran Iran; ^4^ Traditional Medicine and Materia Medica Research Center Shahid Beheshti University of Medical Sciences Tehran Iran; ^5^ Chronic Respiratory Diseases Research Center, NRITLD Shahid Beheshti University of Medical Sciences Tehran Iran

**Keywords:** drug delivery, exosomes, nanoparticles, natural compound, neoplasms

## Abstract

Cancer remains a leading cause of death worldwide, necessitating the development of innovative and more effective treatment strategies. Conventional cancer treatments often suffer from limitations such as systemic toxicity, poor pharmacokinetics, and drug resistance. Recently, there has been growing attention to utilizing natural compounds derived from various sources as possible cancer therapeutics. Natural compounds have demonstrated diverse bioactive properties, including antioxidant, anti‐inflammatory, and antitumor effects, making them attractive candidates for cancer treatment. However, their limited solubility and bioavailability present challenges for effective delivery to cancer cells. To overcome these limitations, researchers have turned to nanotechnology‐based drug delivery systems. Nanoparticles, with their small size and unique properties, can encapsulate therapeutic agents and offer benefits such as improved solubility, prolonged drug release, enhanced cellular uptake, and targeted delivery. Functionalizing nanoparticles with specific ligands further enhances their precision in recognizing and binding to cancer cells. Combining natural compounds with nanotechnology holds great promise in achieving efficient and safe cancer treatments by enhancing bioavailability, pharmacokinetics, and selectivity toward cancer cells. This review article provides an overview of the advancements in utilizing natural substances and nanotechnology‐based drug delivery systems for cancer treatment. It discusses the benefits and drawbacks of various types of nanoparticles, as well as the characteristics of natural compounds that make them appealing for cancer therapy. Additionally, current research on natural substances and nanoparticles in preclinical and clinical settings is highlighted. Finally, the challenges and future perspectives in developing natural compound‐nanoparticle‐based cancer therapies are discussed.

## 1 INTRODUCTION

Cancer is a leading cause of death worldwide, with approximately 19.3 million new cases and 10 million cancer‐related deaths reported in 2020 alone.^1^ Traditional cancer treatments, including chemotherapy, radiation therapy, and surgery have several drawbacks, such as systemic toxicity, poor pharmacokinetics, and drug resistance. Therefore, there is a rising interest in the conception of innovative cancer treatments that are more efficient, less harmful, and better tolerated.^2^, ^3^ Utilizing natural chemicals, which are chemical compounds produced from plants or other naturally occurring sources, is one viable strategy.^4^

Natural compounds are bioactive molecules derived from various sources, including plants, fungi, and marine organisms. Because of their possible therapeutic actions against cancers, these substances have drawn more and more interest in recent years. They have been demonstrated to possess many substances, including antioxidant, anti‐inflammatory, and antitumor action, making them exciting cancer treatment candidates. Furthermore, it has been noted that natural substances are selective toward cancer cells, limiting adverse effects on healthy cells.^5^ Although natural substances have much therapeutic potential, they display some drawbacks that can reduce their effectiveness. Their limited solubility in water is one of their key drawbacks, making it challenging to transport them to cancer cells. Their limited bioavailability, which occurs swiftly during metabolism and excretion from the body before they may reach their target point, is another drawback.^6^

To overcome these limitations, researchers have turned to nanotechnology for drug delivery.^7^ Nanoparticles, carriers with a submicron size, can be created to contain and deliver therapeutic substances to specific locations in the body, including tumors. Compared to traditional chemotherapy, nano delivery has many benefits, such as excellent drug solubility, prolonged drug release, and enhanced cellular uptake, leading to increased efficacy and reduced toxicity.^8^, ^9^ An additional approach to improve drug delivery and specificity involves modifying nanoparticles with specific ligands or carriers, allowing them to recognize and bind to cancer cells accurately. This functionalization enhances the precision of drug delivery and targeting.^10^, ^11^

The synergistic utilization of natural compounds and nano delivery holds great promise in cancer treatment. Encapsulating natural chemicals within nanoparticles can enhance their bioavailability and pharmacokinetics, improving therapeutic effectiveness. Moreover, nano‐delivery methods can potentially minimize the adverse effects of natural substances on healthy cells while enhancing their selectivity toward cancer cells. Combining these two strategies can achieve highly efficient and safe cancer treatments.^12^

Within this context, comprehensive literature searches were performed using Scopus, PubMed, Medline, Embase, and Clinicaltrials.gov to identify publications written in English up to August 2023. Besides, the used keywords were cancer, malignancy, neoplasms, nanoparticles, drug delivery, drug carriers, anthocyanins, ellagic acid, resveratrol, quercetin, nanoparticle drug delivery system, clinical application, clinical trial, exosomes, and extracellular vesicles focusing on the nanoplatforms for delivering natural compounds to cancer cells. We merged founded records and removed the duplicates using EndNote 21 (Thomson Reuters, New York, NY, USA). In order to enhance the precision, three reviewers independently screened the manuscript by title, abstract, and full text to exclude unrelated records. Finally, two expert reviewers extracted data from all qualified studies. This review article provides an overview of current advancements in utilizing natural substances such as anthocyanins, ellagic acid, resveratrol, and quercetin, as well as drug delivery systems based on nanotechnology to treat cancer. We cover the characteristics of natural substances as well as the benefits and drawbacks of various types of nanoparticles for drug administration that make them appealing for cancer therapy. We also highlight current preclinical and clinical research using natural substances and nanoparticles to treat cancer. Finally, we discuss the challenges and future perspectives in developing natural compound‐nanoparticle‐based cancer therapies.

## 2 NANOTECHNOLOGY IN DRUG DELIVERY SYSTEMS

In recent years, nanotechnology has emerged as a promising field with significant potential to develop innovative drug delivery systems for cancer treatment. The application of nanotechnology in medicine offers numerous advantages, including enhanced drug efficacy, targeted delivery, reduced side effects, and improved patient outcomes.^13^

One of the critical challenges in cancer treatment is delivering therapeutic agents to the tumor site while minimizing their impact on healthy tissues. Conventional drug delivery systems often face limitations such as poor solubility, limited stability, and rapid clearance from the body. However, nanotechnology‐based drug delivery systems can overcome these challenges by encapsulating the therapeutic agents within nanoparticles, liposomes, or polymeric micelles.^14^ Nanoparticles, in particular, have gained significant attention due to their unique properties, such as small size, large surface area‐to‐volume ratio, and ability to be functionalized with targeting ligands. These properties enable nanoparticles to accumulate preferentially at the tumor site through passive targeting (enhanced permeability and retention [EPR] effect) or active targeting (ligand‐receptor interactions).^15^ By achieving site‐specific drug delivery, nanotechnology‐based systems can minimize off‐target effects and increase the concentration of therapeutic agents in the tumor, thereby improving treatment outcomes. Furthermore, nanotechnology allows for drug‐controlled release, ensuring sustained and localized drug delivery, which can be achieved by incorporating stimuli‐responsive materials into the drug delivery system. These materials can respond to specific triggers, such as changes in pH, temperature, or enzymatic activity, releasing the therapeutic agent at the desired site.^16^ Additionally, nanotechnology‐based drug delivery systems can facilitate combination therapy by co‐encapsulating multiple drugs or incorporating diagnostic agents within the nanoparticles. This approach allows for synergistic effects, where the combined therapeutic agents act together to enhance efficacy while minimizing drug resistance.^17^

Numerous nanotechnology‐based drug delivery systems have shown promising results in preclinical and clinical studies for cancer treatment. Examples include liposomal formulations of doxorubicin (Doxil®) and paclitaxel (Abraxane®), as well as polymeric nanoparticles carrying docetaxel (Onivyde®). These systems have demonstrated improved pharmacokinetics, reduced toxicity, and increased antitumor activity compared to their conventional counterparts.^18^, ^19^, ^20^ Nanotechnology holds immense potential to revolutionize cancer treatment by overcoming the limitations of traditional drug delivery systems. The ability to achieve targeted and controlled drug release, along with the opportunity for combination therapy, makes nanotechnology‐based drug delivery systems a promising avenue for improving anticancer agents, including drugs derived from natural substances.

## 3 ADVANCEMENTS IN NATURAL SUBSTANCE ENHANCEMENT THROUGH NANOTECHNOLOGY

Natural substances have gained significant attention in cancer treatment due to their potential therapeutic properties. Various natural substances, including anthocyanins, berberin, curcumin, ellagic acid, resveratrol, and quercetin, derived from plants, herbs, marine sources, fungi, and phytochemicals, have shown promise in inhibiting cancer growth and progression. These substances offer diverse compounds with diverse mechanisms of action, providing opportunities for developing novel cancer therapies.^21^, ^22^, ^23^, ^24^ However, they often face disadvantages such as poor solubility, limited stability, and inefficient delivery to the tumor site.^25^

**FIGURE 1:**
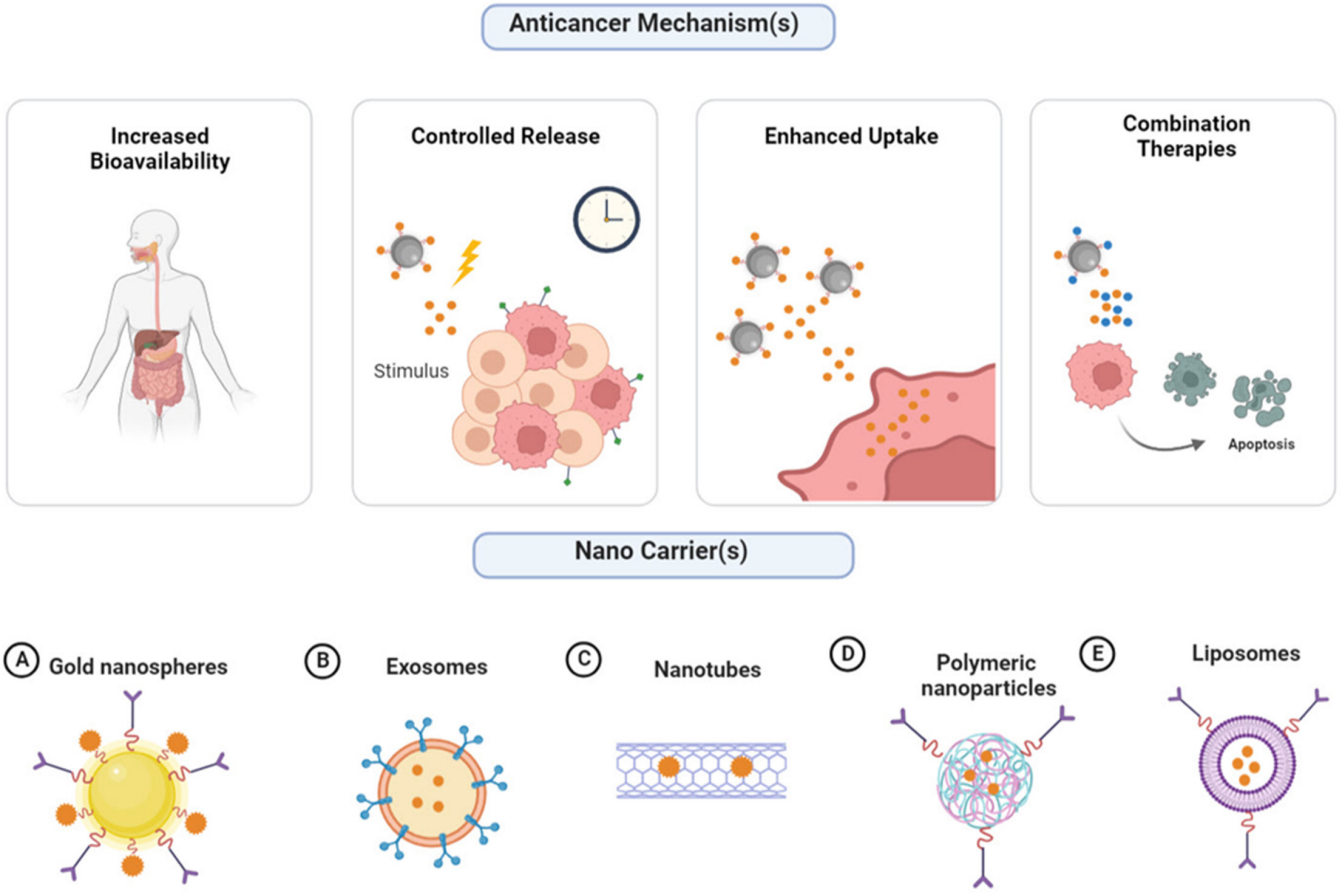
Anticancer mechanisms of Nano drug delivery systems and nano encapsulating agents. One key advantage lies in their ability to increase the bioavailability of drugs, allowing for more efficient and targeted delivery to tumor sites. Additionally, nano drug delivery systems offer controlled release capabilities, enabling sustained drug release over an extended period, thereby optimizing therapeutic efficacy. Another significant benefit is their potential to enhance cellular uptake, facilitating efficient internalization of drugs into cancer cells. Furthermore, these systems can be tailored for combination therapy, allowing for the simultaneous delivery of multiple therapeutic agents to combat different aspects of cancer progression. Encapsulating agents are crucial in Nano‐delivery systems, enabling therapeutic payloads' safe and efficient transport to target sites. Among the diverse range of encapsulating agents, (A) Gold nanospheres, (B) Exosomes, (C) Nanotubes, (D) Polymeric nanoparticles, and (E) Liposomes offer unique advantages and can be tailored to suit specific therapeutic applications, making them valuable components of nano delivery systems. (created by Biorender).

Nanotechnology offers a promising solution to overcome these challenges. Natural substances can be encapsulated and protected by employing nano‐sized carriers such as nanoparticles, liposomes, or polymeric micelles, enhancing their stability and solubility.^14^, ^26^ Additionally, these carriers' surfaces can be improved by targeting ligands to facilitate specific accumulation at the tumor site, improving their efficacy.^27^ Furthermore, nanotechnology allows for the controlled release of natural substances, ensuring sustained and localized drug delivery.^28^ This approach not only enhances their therapeutic effects but also minimizes off‐target effects and reduces toxicity. Nano‐encapsulating agents, along with anticancer mechanisms of nanodrug delivery systems, are represented in Figure 1.

### 3.1 Anthocyanins

**FIGURE 2:**
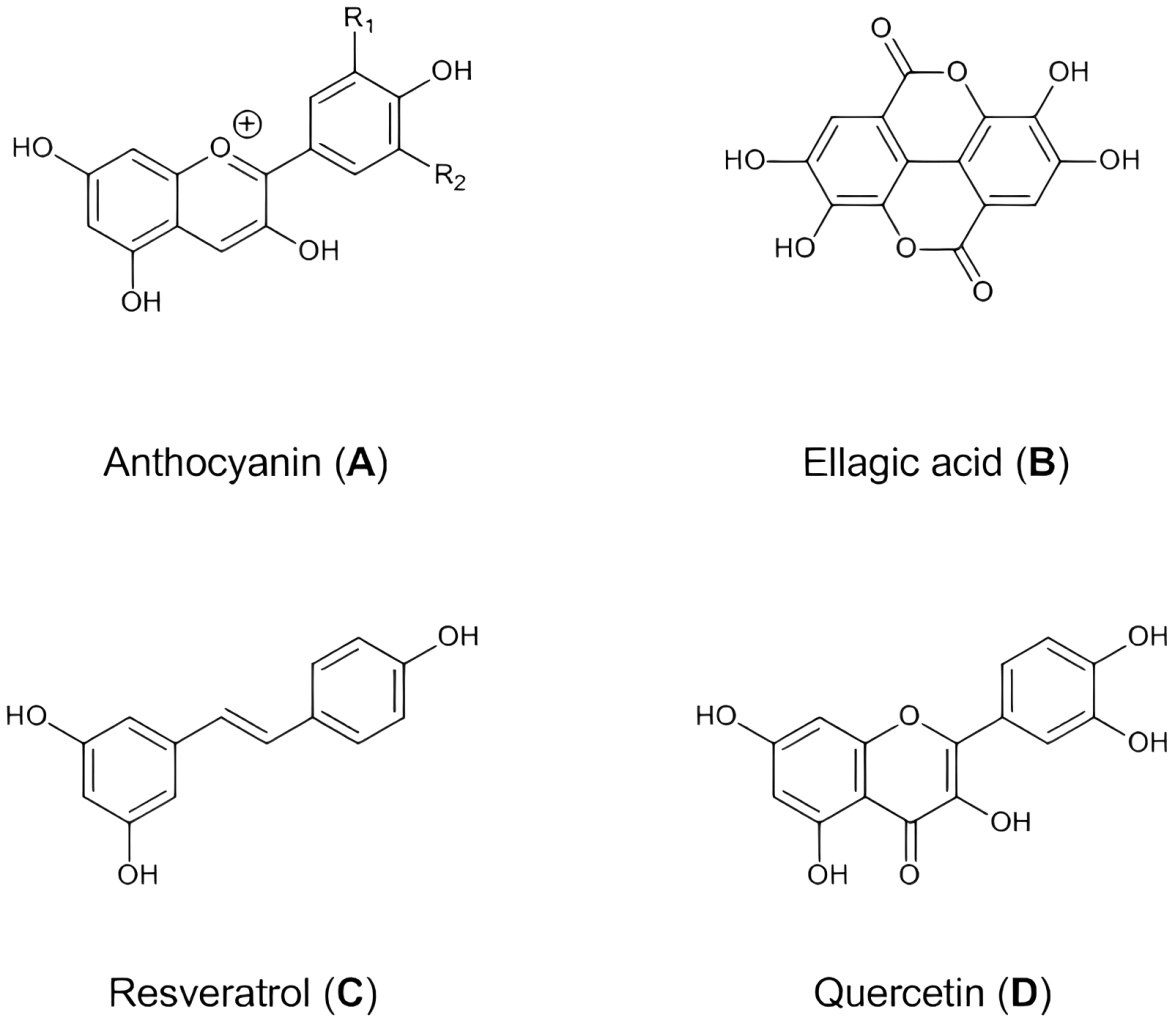
Molecular structure of natural compounds. (A) A C6‐C3‐C6 skeleton primarily characterizes the fundamental structure of anthocyanin. The molecule consists of an anthocyanidin core, hydroxyl groups, sugar moiety, and acyl groups. (B) Ellagic acid is a polyphenolic compound with a molecular formula C14H6O8. It comprises a fused ring system comprising two benzene rings and a lactone ring. (C) The combination of the stilbene backbone, hydroxyl groups, and possible methoxy group defines the molecular structure of resveratrol. (D) The combination of benzene rings, oxygen atoms, hydroxyl groups, double bonds, and the ketone group defines the molecular structure of quercetin.

Anthocyanins are water‐soluble pigments and a significant class of red to blue flavonoids extensively represented in plants. They are responsible for the color properties of many flowers, fruits, and berries and can be used as a natural colorant in food systems.^29^ These compounds are distinguished from other flavonoids due to the presence of flavylium cations and their color change at different pH.^30^ The structure of anthocyanins can be different due to the number and position of hydroxyl and methoxy groups, the type of sugar and their place of substitution (carbon numbers 3, 5, and 7), and the presence of aliphatic or aromatic acids attached to the OH of sugar groups (Figure 2A).^31^

Various research shows that anthocyanins have preventive and therapeutic properties against different cancers. In general, fruits and vegetables containing the natural pigment of anthocyanin have been accepted as preventive agents for colon cancer,^32^ liver cancer,^33^ breast cancer,^34^ prostate cancer,^35^ leukemia,^36^ lung cancer,^37^ and skin cancer.^38^ Since anthocyanins are sensitive to environmental conditions, the biological activity of anthocyanins is significantly reduced during intestinal digestion. Nevertheless, the challenges related to pharmacokinetics, as well as physicochemical drawbacks such as inadequate oral bioavailability, limited permeability, and solubility, have hindered the favorable advancement of this valuable natural pigment as a pharmaceutical product.^39^ Therefore, developing an efficient delivery system of anthocyanins as an anticancer agent is essential.

Using nanotechnology for anthocyanins can protect them from various types of degradation and increase bioavailability for effective therapeutic activity at target sites.^40^ Through encapsulation, anthocyanins can be efficiently delivered to cancer cells, exerting their anticancer effects more effectively while minimizing systemic toxicity. Moreover, encapsulation provides opportunities for combination therapies and synergistic effects with other anticancer agents, reducing chemoresistance. It has been reported that liposomal micelles of bilberry anthocyanin have high stability and bioavailability (more than 90%) in the gastrointestinal (GI) tract. These NutraNanoSpheres were applied to K562 Human Erythroleukemic cancer cells to study apoptotic/cytotoxic effects. The direct cytotoxic effects of NutraNanoSpheres were much higher than those of nonencapsulated bilberry, making it a suitable candidate for clinical trials of cancer patients.^42^ Another study revealed complete tumor ablation after 26 days of anthocyanin treatment in nude mice carrying MCF‐7 under photoacoustic and magnetic resonance imaging with a concentration of 250 μg/mL in polymer nanoparticles. In this study, a detailed evaluation of biodistribution, safety behavior, and elimination of FeAP‐NPs in vitro or in vivo was done. Despite the high accumulation of FeAP‐NPs in the tumor site, a significant amount of these nanoparticles accumulated in the liver due to the increased retention of the reticuloendothelial systems. To isolate FeAP‐NP, deferoxamine mesylate, an iron chelator, was used. This feature is helpful for renal clearance, eliminating the effect of EPR and reducing the accumulation time of FeAP‐NPs in the liver.^43^ Nanoformulation of bilberry anthocyanins has antiproliferative effects on human breast cells MCF7 and MDA‐MB‐231, prostate cells DU145 and PC3, ovarian cells OVCA432, human lung cells H1299 and A549, colon cells HCT‐116 and pancreatic cells Mia PaCa2 and PANC1, after 72 h of treatment and at concentrations from 20 to 100 μM. Lung cancer tumor xenografts in nude mice showed a significant therapeutic response of exosomal anthocyanin compared to free anthocyanin. In many cancers, the NF‐κB signaling pathway is activated by cytokines and interleukins. Using exosomal anthocyanin inhibits NF‐kB activation of cancer cells caused by TNFα.^39^

Controlled release of nano drugs for anticancer agents enables precise and sustained delivery of therapeutic payloads, ensuring optimal drug concentrations at the tumor site while minimizing off‐target effects. This approach enhances treatment efficacy, reduces systemic toxicity, and offers the potential for personalized and targeted cancer therapies. Moreover, controlled release systems based on pH modulation and hydrogen bonding have allowed sustained and precise delivery of anthocyanins to tumor sites, taking advantage of the acidic microenvironment of cancer cells. A significant factor contributing to the sustained release of anthocyanins in a simulated intestinal fluid is the hydrogen bonding between the –OH groups on the inner surface of the nanotubes and anthocyanin molecules. Consequently, the pH of the surrounding environment plays a crucial role in controlling the rate of anthocyanin release. Since cancer cells exhibit higher acidity than normal tissues, utilizing pH‐sensitive halloysite nanotube systems as drug carriers in cancerous tissues is a suitable approach. In this regard, black‐carrot anthocyanins were encapsulated into halloysite nanotubes, and then their cytotoxic effects were evaluated on HT‐29 colon cancer and MCF‐7 human breast cancer cell lines. Cell viability was decreased by a factor of two when comparing the encapsulation of anthocyanins to the use of pure anthocyanins in both cell lines.^40^

Nanoencapsulation has shown considerable effects on drug uptake by cancer cells.^41^ For instance, bilberry anthocyanidins encapsulated with bovine milk–derived exosomes were investigated in colon tumors' chemoprevention.^44^ The outcomes of their study validated that encapsulated anthocyanin exhibits greater antiproliferative characteristics against colon cancer cell lines HCT‐116 and HT‐29. Moreover, the inhibitory concentration (IC50) of encapsulated anthocyanins decreased compared to free anthocyanin. The antiproliferative effects of exosome anthocyanin are more significant and more effective than pure anthocyanin due to increased stability and better cell uptake. Their results showed that encapsulated anthocyanin did not show toxicity in normal colon cells (CCD‐18Co) and only had specific cytotoxicity on colon cancer cells. The administration of anthocyanins to ApcMin/+ mice infected with enterotoxigenic *Bacteroides fragilis* bacteria resulted in a noteworthy decrease in colon tumors.

Combination therapies utilizing nano drugs for anticancer agents hold immense potential in overcoming drug resistance and improving treatment outcomes. Simultaneously delivering multiple therapeutic agents or combining different treatment modalities, such as chemotherapy, immunotherapy, and targeted therapy, these synergistic approaches can enhance cancer cell killing, disrupt the tumor microenvironment (TME), and improve overall therapeutic response. Increasing myelosuppression in mice with HT29 cancer cells by loading cornflower anthocyanins and doxorubicin into hyaluronic acid through hydrophobic and π–π stacking interactions were done by Xiong et al.^45^ Their results showed that this nano complex improved apoptosis and delay of tumor growth of CD44^+^ in HT29 cells of colon cancer. The cytotoxicity of the nano complex was higher compared to doxorubicin alone, and there was a significant increase in the proportion of apoptotic cells. This enhanced effect can be attributed to the specific binding of the anthocyanin nano complex to CD44^+^ and the accelerated release of doxorubicin. Sulfuric hyaluronic acid increases cell endocytosis, and anthocyanins accelerate the release of doxorubicin in the cytoplasm. Also, this nano complex increases the expression of Bax and decreases the expression of Bcl‐2, which plays an essential role in cell apoptosis. In the xenograft model, it was observed that the anthocyanin‐doxorubicin nano complex exhibited higher accumulation at the tumor site in mice. Moreover, the amount of doxorubicin accumulation in the lung decreased, and there was a reduction in its distribution within the kidneys. As a result, this nano‐complex prevents the side effects of doxorubicin.

Nano drugs also hold promise in reducing chemoresistance by bypassing multidrug resistance mechanisms and sensitizing cancer cells to conventional chemotherapeutic agents. Anthocyanin‐loaded nanocarriers have been shown to decrease chemoresistance, inhibit drug efflux pumps, and increase the sensitivity of cancer cells to cytotoxic drugs. Aqil et al. investigated the antiproliferative activity of berry exosome anthocyanins against drug‐resistant and drug‐sensitive ovarian cancer cells at a concentration of 75 μM and their results showed that the growth of OVCA432, OVCA433, A2780/CP70, and A2780 ovarian cancer cells was stopped at this concentration. These exosomal anthocyanins cause a decrease in the effective concentration of cisplatin level needed to inhibit cisplatin resistance of ovarian cancer cells and decrease the expression of glycoprotein P (P‐gp), a known chemoresistant mechanism in cancer cells. In vivo, results showed that anthocyanins increase the sensitivity of human ovarian cancer xenografts to paclitaxel.^46^ Recent findings of the anticancer effects of anthocyanin with nanoparticles are summarized in Table 1.

**TABLE 1:** Studies on the anticancer effects of nanocarrier drug delivery systems with anthocyanins.

Anticancer mechanism	Nano encapsulating Agent(s)	Cancer cell line(s)	Outcome	Ref
Increased bioavailability	‐Liposomal micelles based	K562	‐Cytotoxic effect on K562 human erythroleukemic cancer cells	^42^
	‐FeIII ions ‐Poly (L‐glutamic acid)‐gmethoxy poly(ethylene glycol)	MCF‐7	‐Complete tumor ablation in nude mice carrying MCF‐7	^43^
	‐Bovine milk‐derived exosomes		‐Antiproliferative effects on human breast, prostate, ovarian, human lung, colon, and pancreatic cancer	^39^
Controlled release	‐Halloysite nanotubes	HT‐29 MCF‐7	‐Growth inhibition in cancer cells	^40^
Enhanced uptake	‐Bovine milk‐derived exosomes	HCT‐116 HT‐29	‐Antiproliferative properties on colon cancer cell lines ‐No Cytotoxic effect on normal CCD‐18Co colon cells	^44^
Combination therapies	‐Sulfuric hyaluronic acid (Combined with doxorubicin)	HT‐29	‐Improved apoptosis and delay of tumor growth of CD44^+^ in HT29 cells of colon cancer	^45^
Reduced chemoresistance	‐Bovine milk‐derived exosomes	OVCA432 OVCA433 A2780/CP70 A2780	‐Stop the growth of ovarian cancer cells ‐Decrease in the expression of glycoprotein P	^46^

*Note*: Cancer cell lines: A2780, Ovarian cancer; A2780/CP70, Ovarian cancer; HCT‐116, Colorectal cancer; HT‐29, Colorectal cancer; K56, Chronic myelogenous leukemia; MCF‐7, Breast cancer; OVCA432, Ovarian cancer; OVCA433, Ovarian cancer.

### 3.2 Ellagic acid

Ellagic acid is a polyphenol compound consisting of two lactone rings and four hydroxyl groups (Figure 2B), which is found in a wide range of foods such as nuts and fruits, including strawberry, raspberry, blueberry, cloudberry, walnut, almond, and pomegranate.^47^, ^48^, ^49^ The presence of these hydroxyl groups causes the anti‐carcinogenic activity of ellagic acid.^49^, ^50^, ^51^ Ellagic acid is mainly produced from the hydrolysis of ellagitannins in plants. In addition to free ellagic acid in plants, different derivatives are created due to glycosylation, methylation, and methoxylation.^49^ Ellagitannins are hydrolyzable derivatives of ellagic acid and are not absorbed into the digestive system. After consumption of the ellagitannins through foods, they are hydrolyzed and released as free ellagic acid in the human digestive system.^51^, ^52^ Hydrolysis of ellagitannins is done by gut microbiota or physiological pH, and then free ellagic acid is seen in blood plasma.^50^

Different studies show that ellagic acid has an influential role in the regression of various types of cancer, such as colon cancer,^53^, ^54^ bladder cancer,^55^ lung cancer,^56^ liver cancer,^57^, ^58^ breast cancer,^59^, ^60^ prostate cancer,^61^, ^62^ and endometrial cancer.^63^, ^64^ It induces antiproliferative effects by regulating signaling pathways, inhibiting the cell cycle, and inducing apoptosis.^65^ Despite the anticancer properties associated with ellagic acid, ellagic acid has relatively low bioavailability in the GI tract due to its poor absorption and rapid metabolism, diminishing its ability to inhibit cell proliferation and effectively prevent carcinogenesis within the body. The low bioavailability of ellagic acid is due to poor aqueous solubility and low permeability of the enterocyte epithelial membrane, and it is classified as a class IV drug in the biopharmaceutical classification system.^66^, ^67^ The structural features of ellagic acids and the presence of lactone rings reduce its solubility in the aqueous phase, and on the other hand, the presence of hydroxyl groups reduces lipophilicity and transport throughout the body.^68^, ^69^ Thus, following oral consumption, ellagic acid fails to reach sufficient levels in the plasma and target tissues.^70^

In order to improve the therapeutic effects of this valuable compound, the use of a nanoparticulate delivery system is suggested. Due to its low toxicity and high anticancer activity, ellagic acid encapsulated in hexanuclear metal prisms has been used as a potent anticancer agent against A549 cell lines (lung cancer), AGS (gastric cancer), and SK‐hep‐1 (liver cancer). These metal prisms increase the anticancer activity of the encapsulated compounds by increasing their bioavailability. Encapsulated ellagic acid inhibited the growth of all cell lines and showed the highest cytotoxicity against the A549 cell line. High expression of Rantes (regulated by activation of normal T cells) causes angiogenesis and proliferation in various tumors and, as a result, metastasis. It was shown that encapsulated ellagic inhibits the growth of cancer cells by reducing Rantes secretion and increasing granulocyte colony‐stimulating factor (G‐CSF) secretion.^71^

Nanotechnology enables precise control over drug release, enhanced bioavailability, and targeted delivery to tumor sites. The use of ellagic acid loaded in chitosan nanoparticles for targeted drug delivery in treating oral cancer has been investigated.^72^ Loaded ellagic acid was released after 48 h in laboratory conditions and phosphate buffer medium, showing a sustained drug release pattern. It was found that during the first 3 h, the release rate is rapid, and then the gradual release continues until 48 h. The immediate release is attributed to absorbed ellagic acid on the surface of nanoparticles, and ellagic acid trapped in the polymer matrix causes sustained release. Also, anticancer and antiproliferative studies in the in vitro environment on the KB cell line of human oral cancer showed that ellagic acid shows significant cytotoxicity against cancer cells. Genomic DNA fragmentation confirmed apoptotic cell death by ellagic acid nanoparticles. Although fragmentation was not observed, high DNA damage indicates apoptotic cell death in human oral cancer cells. The reported IC50 value is lower than that of free ellagic acid. Solid lipid nanoparticles have been reported to increase the anticancer activity of ellagic acid in prostate cancer cell line PC3. A burst release was observed in the early hours, followed by a sustained ellagic acid release for up to 72 h. The stability of loaded ellagic acids was good at 4–8°C for 4 weeks. The low IC50 value of loaded ellagic acid was observed in inhibiting cancer cells compared to free ellagic acid. The results of qRT‐PCR showed that ellagic acid loaded into solid lipid nanoparticles upregulated Bax mRNA levels.

Nano‐based technology has emerged as a practical approach for improving the internalization of ellagic acid into cancer cells. The delivery and cellular uptake of ellagic acid can be significantly enhanced through nanoformulations, such as nanoparticles or liposomes. Surface modifications of nanoparticles with targeting ligands or antibodies can further facilitate specific binding and recognition of cancer cells, promoting higher internalization. Cellular uptake and cytotoxicity of chitosan‐ellagic acid films and their effect on inducing apoptotic death of HCT‐116 and Caco‐2 cell lines indicate the cytotoxicity of these nanoparticles.^70^ At the level of in vitro observations, these nanoparticles were able to increase the cytotoxicity against colon adenocarcinoma. Excellent interaction between ellagic acid nanoparticles and cancer cells associated with internalization through rapid nonspecific phagocytosis causes significant arrest in HCT‐116 cell growth. In this study, New Zealand white rabbits were used for in vivo studies and measuring the oral bioavailability of nanoparticles. Their results showed a 3.6‐fold increase in the area under the curve compared to free ellagic acid. Absorption of ellagic acid nanoparticles is carried out through the M‐cells of the lymphoid cells located in the small intestine (Peyer's patches). M‐cells transport the drug to the lymph nodes, and the possibility of hepatotoxicity by the drug is reduced.

Combination therapy involving the synergistic use of ellagic acid with other anticancer agents, coupled with advanced nanotechnology techniques, holds tremendous potential for revolutionizing cancer treatment. Nano‐based drug delivery systems, such as liposomes, polymeric nanoparticles, or dendrimers, offer a platform to encapsulate and co‐deliver ellagic acid and other therapeutic agents. Combined delivery of pemetrexed/ ellagic acid synergistic treatment for breast cancer was performed using lactoferrin (LF) mesoporous silica nanoparticles (MSNs) by Ali et al.^60^ Ellagic acid, which is a hydrophobic compound, is physically placed in the pores of MSNs through the adsorption properties of MSNs. Electrostatic interactions between ellagic acid and MSNs cause ellagic acid entrapment. On the other hand, pemetrexed, which is a water‐soluble compound, is attached to the LF shell through a chemical conjugation. Their results showed that the tumor vessels were disorganized and dilated, and this feature caused the nanocarriers to passively leak from the tumor blood vessels and accumulate in the cancer cells. Also, Lf receptors expressed on the surface of cancer cells interact with MSNPs functionalized with Lf, which ultimately increases cell uptake. Coated LF on MSNs surface controls system toxicity and premature drug release. In comparison to free drugs, the combination of dual drug‐loaded formulations exhibited increased cytotoxicity against MCF‐7 breast cancer cell lines. In another study, researchers developed ellagic acid, albumin, and Fe(III) nanoparticles (EA‐Fe@BSA) designed explicitly for endogenous H2S‐mediated chemodynamic therapy (CDT) in conjunction with photothermal synergy, achieved through enhanced Fe(III)/Fe(II) conversion.^73^ Then, the anticancer effect of these nanoparticles was investigated in vitro and in vivo on HCT116 colon cancer cells and HCT116 tumor‐bearing mice. In this system, Fe(III) catalyzes the conversion of H_2_O_2_ to •OH, which is stronger oxidizing reactive oxygen species (ROS) than H_2_O_2_, thus causing harmful oxidative damage to tumors. The tumor ablation test shows that the efficacy of CDT is synergistically increased by H_2_S and endogenous photothermal therapy (PTT) and significantly suppresses and cures cancer in mice. Enhanced CDT from endogenous reducing agents in tumor cells offers a promising approach to the treatment of colon cancer. Recent findings of anticancer effects of ellagic acid with nanoparticles are summarized in Table 2.

**TABLE 2:** Studies on the anticancer effects of nanocarrier drug delivery systems with ellagic acid.

Anticancer mechanism	Nano encapsulating Agent(s)	Cancer cell line(s)	Outcome	Ref
Increased bioavailability	‐Arene‐ruthenium −1,3,5‐tris(pyridin‐4‐ylethynyl) benzene (tpeb)	SK‐hep‐1 AGS B16/F10	‐ Growth inhibition of SK‐hep‐1, AGS, and B16/F10 cell lines	^71^
Controlled release	‐Chitosan	KB	‐ Significant cytotoxicity on oral cancer (KB cell lines)	^72^
	‐Solid lipid nanoparticles	PC3	‐Reducing IC50 value for PC3 cells	
Enhanced uptake	‐Chitosan	HCT‐116 Caco‐2	‐ Significant arrest in cancer cell growth	^70^
Combination therapies	‐Lactoferrin (LF) mesoporous silica nanoparticles (Combined with pemetrexed)	HT‐29	‐ Increased cytotoxicity against MCF‐7	^45^
	‐FeCl3	HCT‐116	‐Tumor inhibition in HCT116 tumor‐bearing mice. ‐Converting H_2_O_2_ to •OH	^73^

*Note*: cancer cell lines: SK‐hep‐1, hepatocellular carcinoma; AGS, gastric cancer; B16/F10, melanoma; KB, oral cancer; PC3, prostate cancer; HCT‐116, colorectal cancer; Caco‐2, colorectal adenocarcinoma; HT‐29, colorectal cancer.

### 3.3 Resveratrol

Resveratrol (3,5,40‐trihydroxystilbene) is a natural compound categorized as a non‐flavonoid polyphenol (Figure 2C). It significantly addresses ailments triggered by free radicals and oxidative stress, including cancers. In terms of its anti‐carcinogenic properties, it shows effectiveness throughout all steps of cancer development—initiation, promotion, and progression. Resveratrol effectively hinders the growth of various cancer cell types, including breast, skin, liver, colon, prostate, pancreas, lung, and stomach, while also inducing apoptosis.^74^, ^75^ This natural compound induces cancer cell apoptosis via mitogen‐activated protein kinases (MAPK) and protein kinase C (PK‐C) mediated signaling pathways. In addition, it has been reported that resveratrol can reverse multidrug resistance in cancer cells, so its combination with chemotherapeutic drugs such as paclitaxel, doxorubicin, and methotrexate can increase the sensitivity of cancer cells.^76^ Despite the promising anticancer effects of resveratrol, its utilization in clinical and medicinal applications encounters limitations, including inadequate water solubility, easy clearance and instability, and limited bioavailability caused by extensive metabolism in the intestine and liver.

It has been shown that using proper nanocarrier‐based systems like liposomes, polymeric micelles, solid lipid nanoparticles, and polymeric nanoparticles for packaging resveratrol solves this problem by improving bioavailability, drug uptake, sustained release, increasing penetration through biologic membranes, and combination therapy. Studies indicated that resveratrol‐loaded solid lipid nanoparticles (RES‐SLNs) have a superior ability for breast cancer treatment. RES‐SLNs showed strong anti‐proliferation ability and inhibitory effects on the invasion and migration of MDA‐MB‐231 cells. It accelerated the ratio of Bax/Bcl‐2 but reduced the expression of cyclin D1 and c‐Myc.^77^ Studies have indicated an enhancement in the pharmacokinetic function of resveratrol after it was loaded with nanomicelles. In‐vitro anticancer studies displayed that resveratrol‐loaded nanomicelles (RSNM) enhanced the cytotoxic effects against HCT 116 cells (human colorectal cancer cells) rather than the pure resveratrol by inducing apoptosis and improving drug delivery. Also, their pharmacokinetic study showed that the oral bioavailability of resveratrol after oral administration of RSNM is higher than the free resveratrol.^78^

Nanotechnology has revolutionized drug delivery systems by offering a means to enhance the sustained release of resveratrol within tumors. Through the development of nanoparticle‐based formulations, resveratrol can be encapsulated and released in a controlled manner over an extended period. This sustained release mechanism allows for continuous and steady exposure of the tumor cells to resveratrol, maximizing its therapeutic effects. Resveratrol can effectively inhibit growth and induce apoptosis of glioblastoma cancer cells through suppressed STAT3 activation and increased ROS generation, but rapid clearance of resveratrol limited its application in glioblastoma tumors. In this context, glioblastoma‐targeting resveratrol nanoformulation may overcome these therapeutic limitations. An In vivo study by Lin et al. indicated that Pep‐PP^@^ resveratrol nanoparticles (resveratrol encapsulated with IL‐13Rα2‐targeting nanoparticles) have strong cytotoxic effects on rat C6 glioblastoma cells in comparison with free resveratrol, whereas it has little inhibitory effects on normal rat brain cells. Pep‐PP^@^ resveratrol nanoparticles display prolonged resveratrol release (about 25%) in 48 h, and the intracellular residence time of resveratrol in the Pep‐PP^@^ resveratrol group (>24 h) is longer than in the free resveratrol group (<4 h). They reported that resveratrol sustained release improved anti‐glioblastoma effects via triggering c‐Jun N‐terminal Kinase (JNK) and increasing proapoptosis genes expression.^79^ In a study, Marinheiro et al. evaluated the therapeutic effects of resveratrol‐loaded MSNs on human A375 and MNT‐1 melanoma cellular cultures. It has been demonstrated that resveratrol encapsulation with MSNs increased its release in vitro without a burst effect, and the release was faster at acidic pH (pH 5.2). Since the intracellular pH in tumor cells is lower than in normal cells, the pH‐sensitive release behavior of MSNs helps deliver resveratrol to tumor cells. In vitro studies showed that increasing resveratrol‐loaded MSNs concentration decreases the survival of human A375 and MNT‐1 melanoma cellular cultures compared to bulk resveratrol (nonencapsulated). Besides, they have suggested that A375 cells are more sensitive to resveratrol‐loaded MSNs than MNT‐1 cells.^80^ The use of nanoplatforms loaded with resveratrol in the treatment of hepatocytic cancer has been evaluated in several studies. An example is the work by Mohamed G. El‐Melegy et al., who devised innovative nanocochleates nanocarriers loaded with Trans‐Resveratrol (T‐R) to examine their antitumor effects on the HepG2 cell line. Their findings demonstrated a controlled biphasic pattern with prolonged release. Moreover, T‐R loaded nanocochleates enhanced T‐R oral permeability and decreased antiapoptotic (Bcl‐2) and cancer stemness (NANOG) genes compared to free T‐R.^81^

Nanotechnology has emerged as a promising approach to enhance the uptake of resveratrol by cancer cells and crossing biological membranes, including the blood–brain barrier (BBB). By utilizing nanoparticles specifically designed for drug delivery, resveratrol can be encapsulated within these nanocarriers to improve its bioavailability and target cancer cells more effectively. The nanoparticles' small size and surface properties allow for increased cellular uptake, enabling the efficient delivery of resveratrol directly to the tumor site. This enhanced uptake of resveratrol achieved through nanotechnology holds excellent potential for improving the efficacy of cancer treatments and facilitating targeted therapy. D‐α‐Tocopheryl polyethylene glycol succinate resveratrol‐solid lipid nanoparticles (TPGS‐RES‐SLNs) as an effective delivery system promotes the cellular uptake of chemotherapeutic drugs and subsequently increases the efficacy of tumor treatment by inducing apoptosis. In both laboratory (in vitro) and animal (in vivo) studies, it has been discovered that TPGS‐RES‐SLNs effectively suppress the migration and invasion of SKBR3/PR tumor cells while promoting apoptosis. These effects were observed to be more pronounced when compared to free resveratrol without nanoparticle formulation.^82^ Gregoriou et al. reported that resveratrol‐loaded polymeric micelles with a spherical shape and size of 179 ± 22 nm reveal higher uptake efficiency in MCF‐7, MDA‐MB‐231, and MCF‐10A breast cancer cells, resulting in decreased viability of breast cancer cells without any adverse effect on normal cells.^83^ In a separate investigation, researchers successfully loaded resveratrol into Zein nanoparticles, resulting in resveratrol‐Zein nanoparticles (RES‐ZN) with a particle size measuring 137.6 nm and an encapsulation efficiency of 92.3% ± 3.6%. The cellular uptake of RES‐ZN nanoparticles was significantly increased compared to free resveratrol. This compound exerts its anticancer effects in HCT‐116 cells by increasing antiproliferative and proapoptotic markers and inducing oxidative stress. Increased apoptosis is explained by proapoptotic gene expression (CASP3 and cleaved caspase‐3) and decreased expression of antiapoptotic genes (miRNA125b and NF‐κB). RES‐ZN NPs could induce oxidative stress in cancer cells by encouraging ROS formation through the oxidation of NADPH75.^84^

Velaphi C Thipe et al. synthesized a resveratrol‐conjugated gold nanoparticles (RES‐AuNPs) delivery system for applications in cancer therapy. Gum arabic (GA) was used to provide a matrix substrate for increased T‐R loading onto the AuNPs' surface and enhancing the AuNPs' stability. They synergistically investigate gold nanoparticles' inherent proapoptotic characteristics and resveratrol's anticancer properties against MDAMB‐231. RES‐AuNPs showed strong anticancer effects due to enhancing the availability of resveratrol and increasing cellular uptake into tumor cells between 4 and 24 h after treatment.^85^ Resveratrol was successfully loaded into oxidized mesoporous carbon nanoparticles (OMCNs) with high loading efficiency (24.8% w/w). In vitro cytotoxicity and apoptosis tests displayed that resveratrol‐OMCNs increased the cytotoxic and proapoptotic effects through the poly (ADP‐ribose) polymerase (PARP) and Caspase‐3 protein cleavage in triple‐negative breast cancer cells (TNBC). OMCNs demonstrated favorable biocompatibility and exceptional efficiency in cellular uptake.^86^

The combination of resveratrol and conventional chemotherapeutic agents has gained considerable attention in cancer research due to their synergistic effects and potential therapeutic benefits. Based on the evidence, polymeric Nanoparticles (PNPs) have excellent biocompatibility, increased retention time, and targeting capacity properties. In a study, paclitaxel‐resveratrol‐loaded soluplus was fabricated using the thin film hydration method with particle sizes from 102.9 to 945.5. Paclitaxel‐resveratrol PNPs improved BBB penetration and revealed a pooled anticancer effect against the C6 glioblastoma cell line compared to single and combined pure components. Brain distribution and bioavailability of paclitaxel‐resveratrol PNPs compared to pure paclitaxel and resveratrol significantly boosted in mice and augmented anti‐glioma efficacy with enhanced therapeutic component solubility. In addition, this study suggested that the paclitaxel‐resveratrol PNPs cause a reduction of drug release in plasma and can reach the target site.^87^ A study found that when MCF‐7 and CAL‐51 cells (human TNBC lines) were treated with TAM/RES–LbL‐LCNPs, which are nanoparticles that deliver tamoxifen and resveratrol using a layer‐by‐layer approach and liquid crystalline nanoparticle technology, it resulted in the suppression of cell growth and the induction of cell cycle arrest specifically in the G0/G1 phase. The increasing solubility of resveratrol in the structure of the LCNP formula leads to an increased concentration near the cell, and as a result, cytotoxicity rises.^88^ In a separate investigation, researchers developed nanoparticles containing curcumin and resveratrol for targeted and synergistic treatment of hepatocellular carcinoma. Cell and animal studies demonstrated that these nanoparticles, loaded with curcumin and resveratrol, enhance the solubility of both substances, facilitating their absorption by cells. Additionally, the sustained release of the drugs from the nanoparticles prevents rapid metabolism by the liver and kidney, thereby reducing the required dosage. Furthermore, it was proposed that these nanoparticles induce apoptosis in cancer cells by generating ROS and activating caspase 3 in vivo.^89^ A different study involved the development of delivery systems using solid lipid nanoparticles loaded with curcumin‐resveratrol. These SLNs exhibited small mean diameters of 180.17 ± 7.69 nm and possessed a Zeta potential value exceeding −30 mV. They have reported that the in vitro release of Resveratrol is higher compared to curcumin. The skin penetration of SLNs loaded with Curcumin‐Resveratrol was enhanced compared to SLN suspension, causing improvement in RES anticancer efficacy. Moreover, it has been shown that Cur‐Res SLNs synergistically inhibit SK‐MEL‐28 melanoma cell proliferation and cause an improvement in resveratrol anticancer efficacy.^90^ Mohammad Kashif Iqubal et al. developed a lipid nanosystem of 5‐fluorouracil and resveratrol to improve penetration between the epidermis and dermis layers of the skin, thereby aiding in controlling and treating skin cancer. This lipid nanosystem releases drugs uniformly and for a long time, which causes good penetration and distribution of drugs to the dermis layer of the skin. Besides, efficacy experimental indicated lipid nanosystem had greater efficacy on the A431 cell line than the conventional formulation.^91^

Nanotechnology has opened up new avenues for enhancing the targeted‐synergistic effects of resveratrol on tumors. By utilizing nanocarriers and functionalizing them with targeting ligands, resveratrol can be precisely delivered to tumor cells, increasing its concentration at the desired site while minimizing off‐target effects. A study aimed to investigate a promising delivery system for targeted gastric cancer therapy by preparing resveratrol‐modified MSNs (RES‐MSN). The RES‐MSN was successfully fabricated, as confirmed by FTIR and UV analysis. Safety experiments further demonstrated the compatibility of the nanoparticles with normal tissues in animals. The antitumor effects of RES‐MSN were evaluated both in vitro and in vivo. In vitro studies revealed that resveratrol‐loaded MSN exhibited more significant inhibition of proliferation and migration in HGC‐27 and AGS cells (human gastric cancer cell lines) than treatment with resveratrol alone. Moreover, in tumor‐bearing nude mice, the resveratrol‐loaded MSN group showed a more significant reduction in tumor size compared to the group treated with RES alone.^92^ Recent findings of the anticancer effects of resveratrol with nanoparticles are summarized in Table 3.

**TABLE 3:** Studies on the anticancer effects of nanocarrier drug delivery systems with resveratrol.

Anticancer mechanism	Nano encapsulating Agent(s)	Cancer cell line(s)	Outcome	Ref
Increased bioavailability	‐Solid lipid nanoparticles	MDA‐MB‐231	‐Inhibiting invasion and migration	^77^
	‐Nanomicelles	HCT‐116	‐Increasing oral bioavailability ‐Inducing apoptosis	^78^
Controlled release	‐IL‐13Rα2‐targeting nanoparticles (Pep‐PP^@^)	C6	‐Significant cytotoxicity C6 glioblastoma cells	^79^
	‐Mesoporous silica nanoparticles	A375 MNT‐1	‐Maintained resveratrol concentration without an initial burst	^80^
	‐Nanocochleates nanocarriers	HepG2	‐Controlled biphasic pattern with prolonged release	^81^
Enhanced uptake	‐D‐α‐Tocopheryl polyethylene glycol succinate ‐Solid lipid nanoparticles	SKBR3/PR	‐Inducing apoptosis ‐Increased uptake	^82^
	‐ Polymeric micelles	MCF‐7 MDA‐MB‐231 MCF‐10A	‐Decreased viability	^83^
	‐Zein nanoparticles	HCT‐116	‐Increasing antiproliferative and proapoptotic markers ‐Inducing oxidative stress	^84^
	‐Gold nanoparticles	MDAMB‐231	‐Strong anticancer effects	^85^
	‐Oxidized mesoporous carbon nanoparticles	MDA‐MB‐231	‐Increased the cytotoxic and proapoptotic effects	^86^
Combination therapies	‐Polymeric Nanoparticles (Combined to paclitaxel)	C6	‐Preventing tumor progression	^87^
	‐Liquid crystalline nanoparticle (Combined with tamoxifen)	MCF‐7 CAL‐51	‐Suppression of cell growth ‐Induction of cell cycle arrest	^88^
	‐Polymeric nanoparticle (Combined with Curcumin)	HepG2	‐Generating ROS ‐Activating caspase 3	^89^
	‐Solid lipid nanoparticles (Combined with Curcumin)	SK‐MEL‐28	‐Improved skin penetration	^90^
	Lipid nanosystem (Combined to 5‐fluorouracil)	A431	‐Improved penetration between the epidermis and dermis layers	^91^
Targeting therapy	‐Mesoporous silica nanoparticles	HGC‐27 AGS	‐Tumor size reduction	^92^

*Note*: cancer cell lines: MDA‐MB‐231, breast cancer; HCT‐116, colorectal cancer; C6, glioma; A375, melanoma; MNT‐1, melanoma; HepG2, hepatocellular carcinoma; SKBR3/PR, breast cancer; MCF‐7, breast cancer; MCF‐10A, mammary epithelial cells; MDAMB‐231, breast cancer; CAL‐51, breast cancer; SK‐MEL‐28, melanoma; A431, epidermoid carcinoma; HGC‐27, gastric cancer; AGS, gastric cancer.

### 3.4 Quercetin

Quercetin (3,3′,4′,5,7‐pentahydroxyflvanone) is a natural polyphenolic bioflavonoid detected in a wide variety of vegetables, fruits, and grains, including broccoli, kale, berries, onions, apples, oranges, pomegranate peels and tea (Figure 2D).^93^, ^94^, ^95^ It is one of the bioactive components with the most important endogenous antioxidants used extensively in pharmaceuticals, cosmetics, and nutraceuticals.^96^ Quercetin has many biological properties and generally long‐standing anti‐inflammatory capacities. It was also reported that quercetin showed neurological, antiviral, anti‐allergic, and antiangiogenesis effects.^95^, ^97^ Furthermore, research indicated that it has significant anticancer potential. The anticancer activity of quercetin is considered against various types of malignancy involving breast, colorectal, liver, lung, prostate, and skin cancer.^98^ However, administrating quercetin has several drawbacks that cause limitations in pharmaceutical use. This agent is a very hydrophobic phytochemical. Therefore, it has partial solubility in aqueous solutions. Other limitations include GI tract instability and low bioavailability.^99^, ^100^ Moreover, high quercetin doses appear toxic in experimental and clinical studies.^101^ Thus, it is necessary to use new approaches for the pharmacological use of quercetin.^102^

Nanotechnology plays a crucial role in overcoming the drawbacks of using quercetin in cancer therapy by encapsulating this substance within nanoscale delivery systems. These nanoformulations improve the solubility and stability of quercetin, enabling its controlled and targeted release at the tumor site. Additionally, the nanocarriers can protect quercetin from degradation, enhance cellular uptake, and prolong circulation time in the body. Moreover, nanotechnology enables the combination of quercetin with other therapeutic agents, such as chemotherapeutic drugs or targeting ligands, further enhancing its efficacy and specificity against cancer cells. By harnessing the potential of nanotechnology, the drawbacks of quercetin in cancer therapy can be effectively addressed, paving the way for improved treatment outcomes and better patient care.

Combining quercetin with nanomaterials has shown promising anticancer effects by inducing apoptosis and cell death through various intracellular pathways. The specific intracellular pathways involved in this phenomenon vary depending on the cancer type and cellular context but commonly include the activation of caspases, modulation of Bcl‐2 family proteins, disruption of mitochondrial function, and inhibition of various signaling pathways. These findings suggest that the combination of quercetin and nanomaterials holds great potential as a novel strategy for cancer treatment, offering a multifaceted approach to induce apoptosis and eliminate cancer cells via different intracellular mechanisms. Yadav et al. demonstrated that loading quercetin with poly (lactic‐co‐glycolic acid) nanoparticles (PLGA‐QNPs) resolved its low hydrophilicity well and progressed its anticancer possibility. The results showed that PLGA‐QNPs meaningfully reduced cervical and breast cancer cell viability by inducing apoptosis, downregulating PI3K/AKT, and upregulating FoxO1, Caspase‐3, and 7.^100^ Researchers used quercetin‐encapsulated chitosan functionalized copper oxide nanoparticles (CuO‐ChNPs‐Q) as another new nano‐drug system for cancer therapy. The in vitro results of CuO‐ChNPs‐Q verified the potent anticancer activity of the CuO‐ChNPs‐Q. The in vivo data showed that CuO‐ChNPs‐Q significantly reduced breast tumor progression in rats by increasing p53 gene expression, caspase‐3, and cytochrome c. Also, it can arrest the cell cycle and suppress proliferative genes such as the PCNA gene.^103^ Another targeted drug delivery system for lung cancer is MSNs (SBA‐15). The biological examination of the A549 cancer cell line indicated the higher efficacy of Q‐SBA‐15 than quercetin alone. They showed that the PI3K/AKT/mTOR pathway acts as the apoptotic signaling in A549 cells.^104^

Titanium dioxide nanotubes (TNT) have more significant surface space for carrier particles with exceptional physical and chemical features. The effect of conjugated TNT with quercetin (TNT–Qu) on the B16F10 animal melanoma model proved that topical TNT–Qu could be applied to reduce tumor growth via regulating phospho‐STAT3 levels in the tumor. The histopathological results on treated squamous cell carcinoma indicated reduced hyperplasia and inhibition of blood vessel development.^96^ In the study of Ramalingam et al., the zinc oxide (ZnO) nanoparticles were produced and functionalized with quercetin (ZnO‐Quercetin) for ovarian cancer treatment. In vitro experiments showed exceptional activity by creating intercellular oxidative stress and depolarizing mitochondrial membrane potential in ovarian teratocarcinoma cell line PA‐1. The dual staining examination displayed that the ZnO‐Quercetin induces late apoptosis over activation of the intrinsic apoptosis signaling pathway in ovarian cancer cells.^105^ The synthesized quercetin‐caffeic‐acid phenethyl ester (CAPE)‐co‐loaded (PLGA) nanoparticles (QuCaNP) are novel platforms for colorectal carcinoma. Data demonstrated that QuCaNP increased the protein expression of the intrinsic apoptosis pathway, such as caspase‐9 and caspase‐3, in the human HT‐29 cell line.^106^

Nanotechnology plays a crucial role in the administration of quercetin, particularly in enhancing its efficacy by enabling extended and consistent release within the TME. This controlled release ensures a sustained supply of quercetin within the TME, maximizing its therapeutic potential. Furthermore, nanotechnology facilitates targeted delivery, enabling the accumulation of quercetin specifically at the tumor site while minimizing off‐target effects and reducing systemic toxicity. Recently, a mixed micellar system including PF‐127 and Tween 80 was made to increase quercetin's loading content. Quercetin‐loaded mixed micelles were produced by the thin‐film hydration method. Then, the therapeutic efficiency of nano‐micelles as a drug delivery system for breast cancer was investigated. The outcomes of drug release experiments conducted in a laboratory indicated that the mixed micellar system demonstrates a more extended and consistent release pattern when compared to the free quercetin solution. Besides, it exhibited a lower half‐maximal IC50 than the free drug.^107^

Nanoparticles can take a role in improving the administration of quercetin by enhancing its uptake within cancer cells. Nanocarriers can be engineered with surface modifications that facilitate specific interactions with cancer cell receptors, promoting efficient uptake of quercetin. Additionally, the small size of nanoparticles enables them to penetrate cellular barriers more effectively, allowing for enhanced cellular uptake of quercetin.^108^ Ganthala et al. found synergistic effects of combining erlotinib and quercetin against NCI H460 and A549 cells. These nanoparticles can reduce nuclear epidermal growth factor receptor (nEGFR) and P‐glycoprotein (P‐gp) expression resulting in resistance reduction. Moreover, apoptosis induction and drug uptake were increased in A549/ER cancer cells. In addition, in vivo results demonstrated that the uptake of nanoparticles in the lung tissue was raised, and considerably nEGFR expression was reduced.^109^

Nanotechnology improves quercetin administration, especially when combined with other anticancer agents and approaches. Incorporating quercetin into nano‐sized delivery systems, such as nanoparticles or liposomes, allows it to co‐deliver multiple therapeutic agents concurrently. This synergistic combination enhances the anticancer effects by targeting multiple pathways and improving therapeutic outcomes. In the study of Mekkawy et al., letrozole‐(LTZSPs) and quercetin‐loaded spanlastics (QuSPs) using diverse activators such as Brij 35, Cremophor RH40, and Tween 80 were prepared against MCF‐7 cells. Moreover, evaluation of the in vitro analysis showed that cytotoxicity and intracellular ROS of the combination therapy were superior to the single treatments and the soluble free drugs against breast cancer cells.^110^ Quercetin, Scorpion venom peptides (SV), and Phospholipon® 90H (PL) were assimilated in a nano‐based delivery system to evaluate quercetin antiproliferative ability against human breast cancer cells MCF‐7. Cell cycle examination discovered that treatment with this system resulted in substantial cell cycle arrest at the S phase. Also, increased caspase‐9, Bax, Bcl‐2, and p53 mRNA expression in quercetin treatment was observed.^98^ Tiwari et al. investigated the combination of quercetin and gefitinib loaded onto graphene oxide (GO‐PVP) nano vehicle as an anticancer treatment. The cytotoxicity of PA‐1 ovarian cancer cells after the combined system treatment was significantly higher than drugs alone.^111^ Gallic acid and quercetin in the chitosan nano platform are used for colorectal cancer therapy. MTT assay showed that this nano‐drug is cytotoxic on the HCT 116 cell line. Further advantages included reducing antioxidant enzymes, including colonic catalase, glutathione, and superoxide dismutase levels.^94^ Askar et al. used iron oxide nanoparticles as a drug delivery platform in another study. They formulated quercetin‐conjugated magnetite nanoparticles (QMNPs) via simple organic nanoprecipitation. The in vitro studies indicated that MCF‐7 cancer cells incubated with QMNPs inhibited the proliferation and development of cancer cells. The concurrent utilization of QMNPs and irradiation effectively inhibited the proliferation of MCF‐7 cancer cells and resulted in noticeable morphological alterations. The in vivo result focusing on the chronic toxicity of QMNPs revealed no toxic influence on renal, hepatic, and hematological markers. Furthermore, QMNPs blocked tumor growth in albino rats using immunomodulation, apoptosis induction, and cell cycle arrest.^101^ Recent findings of the anticancer effects of quercetin with nanoparticles are summarized in Table 4.

**TABLE 4:** Studies on the anticancer effects of nanocarrier drug delivery systems with quercetin.

Anticancer mechanism	Nano encapsulating Agent(s)	Cancer cell line(s)	Outcome	Ref
Increased bioavailability	‐Poly (lactic‐co‐glycolic acid) nanoparticles	HeLa MCF‐7	‐Reducing cervical and breast cancer cell viability ‐Inducing apoptosis ‐Downregulating PI3K/AKT, ‐Upregulating FoxO1, Caspase‐3, and 7	^100^
	‐Chitosan ‐Copper oxide nanoparticles	HCT‐116	‐Increasing p53, caspase‐3, and cytochrome c ‐ Cell cycle arrest	^103^
	‐Mesoporous silica nanoparticles	A549	‐Inducing apoptosis by PI3K/AKT/mTOR pathway	^104^
	‐Titanium dioxide nanotubes	B16F10	‐Reduced hyperplasia ‐Inhibition of blood vessel development	^96^
	‐Zinc oxide nanoparticles	PA‐1	‐Intercellular oxidative stress ‐Depolarizing mitochondrial membrane potential	^105^
	‐Caffeic‐acid phenethyl ester ‐Poly (lactic‐co‐glycolic acid) nanoparticles	HT‐29	‐Inducing intrinsic apoptosis pathway	^106^
Controlled release	‐Micellar system (PF‐127 and Tween 80)	MCF‐7	‐Reducing IC50	^107^
	‐Spanlastics	MCF‐7	‐Increased cytotoxicity ‐Intracellular ROS ‐Sustained drug release for 24 h	^110^
Enhanced uptake	‐Solid lipid nanoparticles	NCI H460 A549 cells	‐Increased uptake in lung tissue	^109^
Combination therapies	‐Solid lipid nanoparticles (Combined with erlotinib)	NCI H460 A549 cells	‐Reducing drug resistance ‐Reducing p‐gp expression	^109^
	‐Spanlastics (Combined to letrozole)	MCF‐7	‐ ncreased cytotoxicity ‐Intracellular ROS	^110^
	‐Phytosomes (Combined to venom peptides (SV) and Phospholipon® 90H (PL))	MCF‐7	‐Cell cycle arrest ‐Inducing apoptosis ‐Reduced activity of TNF‐α and NF‐κB	^98^
	‐Graphene oxide (Combined with gefitinib)	PA‐1 IOSE‐364	‐Improved cytotoxic effects	^111^
	‐Chitosan (Combined to Gallic acid)	HCT‐116	‐Reduction of antioxidant enzymes ‐Improved cytotoxic effects	^94^
	‐Iron oxide nanoparticles (Combined with irradiation)	MCF‐7	‐Blocked tumor growth	^101^

*Note*: cancer cell lines: HeLa, cervical cancer; MCF‐7, breast cancer; HCT‐116, colorectal cancer; A549, lung cancer; B16F10, melanoma; PA‐1, ovarian cancer; HT‐29, colorectal cancer; NCI H460, lung cancer; IOSE‐364, immortalized ovarian surface epithelial cells.

## 4 CLINICAL APPLICATIONS

Clinical implementation of natural nanoparticle‐based cancer treatments is essential in their development. However, the complex nature of nanoparticles and the diversity of natural compounds can complicate these therapeutics' regulatory approval process. In addition, the high cost of clinical trials can also be a significant obstacle to clinical implementation.

Despite these challenges, the future of cancer therapy based on natural compounds and nanoparticles is promising. Developing more efficient and stable nanoparticle formulations and using predictive preclinical models may accelerate the clinical translation of these therapeutics. Moreover, combining natural compounds with other therapeutic modalities, such as chemotherapy and immunotherapy, may enhance their efficacy and broaden their clinical application. Ultimately, developing natural compound‐nanoparticle‐based cancer therapies may provide a more effective and targeted approach to cancer treatment. Hence, a better understanding of the anticancer mechanisms of natural compounds and a more investigation of the crosstalk between NP‐based drug delivery systems and tumor metabolism are warranted for drug development and utilization.^112^

A few clinical studies have been registered to treat cancer using natural compounds based on nanoparticles. A phase 2 clinical trial (NCT05456022) has been launched (2022) to investigate the anticancer effect of quercetin‐free or encapsulated by NPs on the tongue squamous cell carcinoma (TSCC) cell line. This study uses three therapeutic approaches including quercetin encapsulated in polyethylene glycol‐polylactide‐co‐glycolic acid (PEG‐PLGA) nanoparticles, quercetin 3,3′,4′,5,6‐Pentahydroxyflavone, 2‐(3,4‐Dihydroxyphenyl)‐3,5,7‐trihydroxy‐4H‐1‐benzopyran‐4‐one, and doxorubicin as a positive control. They will evaluate the viability of SCC cells, BCL‐2, BAX, and PI3K gene expression. Besides, a phase 1 clinical trial, NCT05736224, was recently designed to evaluate the effects of a novel sunscreen formulation for preventing skin cancer. The research team has developed a bioadhesive nanoparticle (BNP) sunscreen that encapsulates avobenzone and octocrylene to prevent the penetration of organic UVR filters into the skin. Additionally, nontoxic natural products, including T‐R, have been integrated into this sunscreen to enhance its UVR absorption capacity, thereby reducing oxidative damage and indirect DNA damage. This study will test the ability of this organic sunscreen to prevent direct and indirect cellular and DNA damage in human skin exposed to ultraviolet radiation. To the best of our knowledge, there are a few clinical trials of nano‐delivery of anticancer natural components for preventive and/or therapeutic purposes; therefore, further studies are needed to improve translational insight into anticancer natural components.

## 5 CHALLENGES AND FUTURE PERSPECTIVES

Natural compounds have been extensively explored as potential anticancer agents due to their capacity to selectively target cancer cells while sparing normal cells.^113^ However, these substances' poor solubility, low bioavailability, and quick bodily elimination typically restrict their therapeutic usefulness. Natural compounds have been formulated into nanoparticles, which can increase their stability, solubility, and bioavailability to get around these problems.^114^ Natural compound‐nanoparticle‐based cancer treatments have become a potential strategy for treating cancer in this setting. However, some obstacles must be overcome in developing natural compound‐nanoparticle‐based cancer therapeutics. One of them is choosing suitable natural materials. Although natural compounds have been shown to have anticancer properties, not all of them are suitable for nanoparticle formulation. Therefore, selecting suitable natural compounds that can be efficiently loaded into nanoparticles and exhibit potent anticancer activity is essential.^115^ Another challenge is to optimize the formulation of nanoparticles. Considering the intricate nature of tumors, optimizing the physicochemical attributes of nanocarriers, such as morphological features, size, surface electronegativity, and hydrophobicity, can enhance the efficacy of co‐delivery systems. Introducing ligands to modify nanocarriers further improves targeting efficiency.^116^ In this regard, a novel approach combines natural products, immunotherapy, radiotherapy, gene therapy, photodynamic therapy, PTT, and magnetic hyperthermia.^117^ However, the novel direction may be exploring research avenues related to co‐packaged drugs and/or separately packaged drugs, considering various pharmacokinetics and pharmacodynamics of the drugs. Suggesting revolutionary delivery routes is another pivotal challenge in the field of nano‐drugs. Intravenous injection stands out as the predominant method for administering cancer treatment, involving the introduction of drugs into the patient's venous vessels, allowing them to reach the affected area through systemic circulation. However, recent years have witnessed the emergence of alternative administration routes, opening up innovative possibilities for cancer therapy. One such approach involves pulmonary administration, specifically for the in‐situ treatment of lung cancer through inhalation. In comparison to the traditional systemic administration via vein injection, pulmonary inhalation treatment offers advantages such as reduced drug dosage and minimized toxic side effects throughout the entire body. This method holds significant promise for development, particularly in delivering macromolecule drugs with low bioavailability.^118^

Preclinical evaluation is another critical issue that should be done before using the natural compounds‐nanoparticle‐ based oncolytic agents. Health authorities responsible for approving safe and effective medicines face challenges posed by the emergence of products based on new technologies. Therefore, preclinical evaluation of cancer therapies based on natural compounds and nanoparticles is necessary to assess their efficacy, safety, and pharmacokinetics. Since preclinical evaluation is time‐consuming and expensive, and animal studies are generally not reproducible or reliable, the development of more accurate and standardized evaluation of toxicities in preclinical models and human clinical trials may be critical in predicting the safety and efficacy of these novel therapeutic agents.^119^

## 6 CONCLUSION

Nanotechnology has emerged as a powerful tool in developing innovative drug delivery systems for cancer treatment. The application of nanotechnology offers significant advantages, such as enhanced drug efficacy, targeted delivery, reduced side effects, and improved patient outcomes. Nanoparticles and other nanocarriers provide a means to overcome the limitations of conventional drug delivery systems, enabling site‐specific drug delivery, controlled release, and combination therapy. In the context of natural substances, nanotechnology‐based systems can enhance their solubility, stability, and delivery to the tumor site, unlocking their full therapeutic potential. Integrating nanotechnology with natural substances presents a promising avenue for developing more effective and targeted cancer therapies. Continued advancements in this field promise to improve cancer patients' treatment outcomes and quality of life.

## AUTHOR CONTRIBUTIONS

**Asma Manzari‐Tavakoli:** Project administration (equal); writing – original draft (equal); writing – review and editing (equal). **Amirhesam Babajani:** Methodology (equal); project administration (equal); writing – original draft (equal); writing – review and editing (equal). **Maryam Manzari Tavakoli:** Methodology (equal); writing – original draft (equal). **Fahimeh Safaeinejad:** Writing – original draft (equal). **Ameneh Jafari:** Conceptualization (lead); project administration (equal); supervision (lead); writing – original draft (equal); writing – review and editing (equal).

## FUNDING INFORMATION

No funding.

## CONFLICT OF INTEREST STATEMENT

None declared.

## ETHICS STATEMENT

Not applicable.

## CONSENT

Not applicable.

## Data Availability

No data was used for the research described in the article.
